# BD Vacutainer™ Urine Culture & Sensitivity Preservative PLUS Plastic Tubes Minimize the Harmful Impact of Stressors Dependent on Temperature and Time Storage in Uropathogenic Bacteria

**DOI:** 10.3390/jcm13175334

**Published:** 2024-09-09

**Authors:** Samuel Treviño, Eduardo Ramírez-Flores, Steffany Cortezano-Esteban, Hugo Hernández-Fragoso, Eduardo Brambila

**Affiliations:** 1 Laboratory of Metabolomic and Chronic Degenerative Diseases, Physiology Institute, Meritorious Autonomous University of Puebla, Prol. de la 14 Sur 6301, Ciudad Universitaria, Puebla C.P. 72560, Mexico; hugo_hdezfragoso@hotmail.com; 2 Center for Care and Research in Health Services, Urinalysis and Microbiology Area, Rio Nexapa 6153, Col. San Manuel, Puebla C.P. 72560, Mexico; 33zinc@gmail.com (E.R.-F.); steffany_coes@hotmail.com (S.C.-E.); 3 Laboratory of Chemical-Clinical Investigations, Department of Clinical Chemistry, Chemistry Department, Meritorious Autonomous University of Puebla, 14 Sur. FCQ1, Ciudad Universitaria, Puebla C.P. 72560, Mexico; eduardobrambila1@yahoo.com.mx

**Keywords:** uropathogens, reactive oxygen species, bacteria stressors, viable but non-culturable state, urine culture preservatives

## Abstract

**Background:** Urinary tract infection is a worldwide health problem. According to the Clinical Laboratory Improvement Amendments and the European Urinalysis Guideline, urine samples should be tested within 2 h of collection. Thus, using chemical preservatives that guarantee the pre-analytical conditions is a practical tool. However, the effects of temperature and storage time as uropathogenic bacteria stressors are unclear. **Methods:** Gram-negative and -positive ATTC strains, *E. coli*, *P. mirabilis*, *E. faecalis*, and *S. aureus*, were used in this study. Strains in liquid media were stored at 4, 25, and 37 °C for 0, 2, 12, 24, and 48 h in tubes with and without preservatives. Then, reactive oxygen species (ROS) levels, viable but non-culturable bacteria (VBNC), and bacteria growth were analyzed. **Results:** A high ROS level was associated with the presence of VBNC and dead bacteria with low CFU counts, but a low ROS level increased the CFU number, depending on temperature and storage time in tubes without preservatives (boric acid, sodium borate, and formate). The BD Vacutainer™ Urine Culture & Sensitivity Preservative PLUS Plastic Tubes (C&S-PP) prevent this ROS increase, maintaining the CFU number for longer. **Conclusions:** C&S-PP tubes minimize the stressor effects (temperature and time storage) on uropathogenic bacteria when stored, improving the pre-analytical conditions of cultures realized by the clinical laboratory.

## 1. Introduction

Urinary tract infections (UTIs) are a prevalent global health concern. Diagnosis relies on a combination of clinical symptoms and laboratory confirmation through urine culture [1]. Urine cultures represent a significant workload for clinical laboratories [2]. Working with freshly collected urine specimens is mandatory to avoid contaminant organisms growing and affecting the initial number of uropathogens emitted in primary specimens. Ideally, urine samples should be analyzed within 2 h of collection to minimize the growth of contaminant organisms and ensure accurate results. However, delays in transportation to reference laboratories present a challenge [3]. In cases of inevitable delays, two main strategies are employed to maintain sample integrity: (a) refrigeration, a method that slows bacterial growth but lacks a standardized storage duration due to variable urine composition; and (b) chemical preservatives, which offer a controlled environment for bacterial stability, allowing analysis up to 24 h later [4].

Time, temperature, and urinary constituents can be bacterial stressors. Therefore, storage or maintenance conditions must be studied [5]. Bacteria encounter various environmental stresses in nature, including temperature changes, nutrient limitations, and oxidative stress. They adapt to these stresses through complex mechanisms involving gene expression, protein activity, and cellular metabolism [6,7]. These responses can differ between Gram-positive and Gram-negative bacteria [6]. Reactive oxygen species (ROS) have been proposed to play critical roles in microbial metabolism and responses to environmental stressors [8].

Two of the most widely studied forms of ROS, superoxide and hydrogen peroxide, are constantly produced endogenously through the autoxidation of molecular oxygen [9]. While low ROS levels can promote survival, high levels can be lethal by causing DNA damage, protein dysfunction, and lipid peroxidation [10]. Bacteria possess antioxidant enzymes like superoxide dismutases, catalase, and peroxidases to combat ROS and mitigate their harmful effects [11]. However, when exposed to extreme ROS stress, bacteria may enter a viable but non-culturable state (VBNC) [12,13].

VBNC bacteria are alive but cannot be detected by conventional culture methods due to their inability to grow and form colonies. This poses a diagnostic challenge, particularly in urine samples with low colony-forming units (CFUs) due to inadequate preservation or high ROS levels [13]. The VBNC stage has been described and documented for Gram-negative and positive bacteria. The capacity of bacteria to enter the VBNC stage is associated with different environmental stressors, such as temperature, time analysis, metabolic rate, nutrient deficiency, and toxic metabolites in the primary specimens [14,15]. The VBNC stage generates contradictory results between microscopy analysis and bacterial cultures. Although the VBNC stage is reversible, concerns exist, especially for uropathogenic bacteria, because switching to the infectious stage can happen again in the host once the stressor diminishes or disappears. Therefore, in this study, we aimed to investigate the effects of storage temperature and time on the recovery of ATCC Gram-negative and -positive strains without preservatives compared to BD Vacutainer™ Urine Culture & Sensitivity Preservative PLUS Plastic Tubes (C&S-PP).

## 2. Material and Methods

### 2.1. ATCC Strains

Four ATCC strains were obtained from the microbiology department of the Benemeritous University of Puebla, Mexico: two Gram-negative, *Escherichia coli* (25922) and *Proteus mirabilis* (43071); and two Gram-positive, *Enterococcus faecalis* (29212) and *Staphylococcus aureus* (25923) (Thermo Scientific™ Culti-Loops™, Thermo Fisher Scientific Inc; Waltham, MA, USA). Strains were rehydrated in liquid media, Culti-Loops Aerobic Set Rehydration Fluid (Thermo Fisher Scientific Inc; Waltham, MA, USA). Biochemical characterization was realized according to their certificates of analysis. Then, we used the McFarland procedure to adjust two groups of three sets of five aliquots of 10 mL of each strain in aerobic set rehydration fluid to 1.0 × 10^6^ cells/mL. The first work group was placed in C&S-PP tubes, and the second work group was placed in sterile conical tubes of 15 mL with hermetic cover without preservatives. Each aliquot set was incubated at 4 °C, 25 °C, and 37 °C for 0, 2, 12, 24, and 48 h, respectively. After incubation, all samples were analyzed in the VBNC state using the acridine orange method and for ROS production using the DCFH-DA method. Then, they were inoculated onto quintuplicate Trypticas Soy Agar (TSA; Becton Dickinson, Sparks, MD, USA) plates with 5% sheep blood, MacConkey agar (only for Gram-negative strains; Becton Dickinson, Sparks, MD, USA), and Mannitol Salt Agar (only for *S. aureus*; Becton Dickinson, Sparks, MD, USA), 0.01 mL with an inoculation loop. The media plates were incubated at 37 °C for 24 h, except for *P. mirabilis*, which was read at 16 h to facilitate colony counting and avoid swarming development. The bacterial counting and recording were as colony-forming units per milliliter (CFU/mL) from each plate.

### 2.2. Reactive Oxygen Species Quantification

A fluorometric method was used to quantify ROS levels in the samples. A total of 50 μL of each strain in aerobic set rehydration fluid was mixed with 450 μL of 40 mM TRIS plus HEPES buffer and then incubated with 5 μM of 2′7′-dichlorodihydrofluorescein diacetate (DCFH-DA). The samples were incubated in the dark for 20 min at 37 °C under constant shaking. Fluorescence signals were determined in a PerkinElmer LS50-B luminescence spectrometer at 488 nm excitation and 525 nm emission wavelengths. Values were obtained by interpolating the readings with a 2′7′-dichlorofluorescein (DCF) standard curve (Sigma Aldrich, St. Louis, MO, USA) [15].

### 2.3. Viable but Non-Culturable Bacteria Assay

Acridine orange (AO) staining was used to assess VBNC bacteria. A work solution was prepared with 2 mg/mL AO in distilled water diluted to 1:100 with a 10 mM citrate-phosphate buffer, 0.1 M NaCl at pH 3.8 (to 150 mL of distilled water, 9.92 mL of 0.1 M Citric Acid, 5.46 mL 0.2 M of dibasic sodium phosphate, and 1.7 g of NaCl were added). Then, on sterile slides were placed 25 µL of each sample, 25 µL work solution was added, and this was agitated for 5 min at room temperature. A coverslip was set, and viability was evaluated using a fluorescence microscope attached to a camera (Leica Microsystems GmbH, Wetzlar, Germany). All reagents used were acquired from Sigma-Aldrich (Sigma-Aldrich, St. Louis, MO, USA). AO interacts with DNA differently in viable, nonviable, and VBNC bacteria. Viable bacteria intercalate DNA with AO, emitting green fluoresces (525 nm), while nonviable or dead bacteria emit red fluoresces (>630 nm), and VBNC bacteria emit yellow to orange fluoresces [16].

### 2.4. Statistical Analysis

A Shapiro–Wilk normality test was performed to verify that the different data come from a normally distributed population. The results were expressed as the mean ± SEM for all experiments. Two-way ANOVA followed by Bonferroni tests were used to analyze the effects of storage time, preservative presence, and their interaction on bacterial growth, informed as F, using GraphPad Prism 8 (GraphPad Software Inc., USA). (*) The significance level was set at *p* ≤ 0.05, taking the time zero as a reference. Multiple regression analysis was performed using SPSS 22.0 software (SPSS Corporation, Chicago, IL, USA) to assess the relationships between preservatives, temperature, time, and ROS on bacteria growth, with a significance level of *p* ≤ 0.05 [17].

## 3. Results

**Figure 1:**
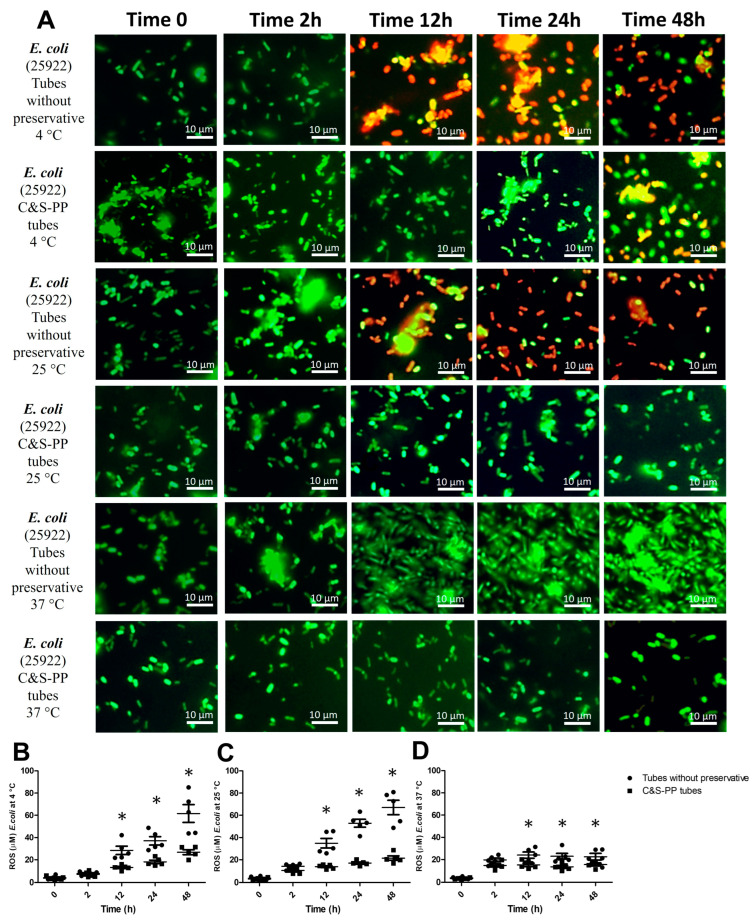
**ROS levels and VBNC *E. coli* at storage times and temperatures with and without preservatives.** (**A**) *E. coli* viable but non-culturable (VBNC); (**B**) *E. coli* ROS level at 4 °C; (**C**) *E. coli* ROS level at 25 °C; (**D**) *E. coli* ROS level at 37 °C. The results shown are the average of five different experiments ± SEM. (*) A two-way ANOVA followed by Bonferroni tests indicates a significant difference regarding the control time, zero minutes (*p* ≤ 0.05). **ROS**, Reactive Oxygen Species.

The maintenance of four bacteria strains preserved in BD Vacutainer™ Urine C&S-PP tubes was studied. ROS generation was evaluated because its excess could interfere with the growth of bacteria. This first valorization was realized considering the independent variables such as temperature, time, and preservative use. The results showed that the preservative effect on ROS generated by *E. coli* at 4 °C was 14.4% of the total ROS variance (F = 39.3). Meanwhile, time conservation affects the result by 61.6% of the total ROS variance (F = 54.2). ROS concentration increased by 118.8% (12 h), 150% (24 h), and 138.8% (48 h) in tubes without preservatives (Figure 1B). The ROS increase is associated with the presence of VBNC bacteria (yellow and orange fluorescence) and dead bacteria (red fluorescence); however, bacteria in C&S-PP tubes observed viability until 24 h (green fluorescence, Figure 1A).

At 25 °C, the preservative effect on ROS was 25.9% (F = 228.3), and time conservation affected the result by 49.4% of the total ROS variance (F = 58.9). The ROS concentration increased similarly to the previous temperature analyzed (Figure 1C). However, the VBNC state was only observed at 12 h, while at 24 and 48 h incubation, dead bacteria predominated in tubes without preservatives, and viable bacteria were observed in C&S-PP tubes until 48 h (Figure 1A). Interestingly, the conservation at 37 °C was only 15.2% (F = 12.5) from the preservative effect on ROS, and time conservation affected the result by 58.7% of the total ROS variance (F = 38.7). Although the ROS concentration was significantly high from 12 to 48 h (105%, 68.6%, and 42.7%), it was below the previous temperatures analyzed (Figure 1D). Due to this, a significant number of viable bacteria were observed in tubes without preservatives (Figure 1A).

**Figure 2:**
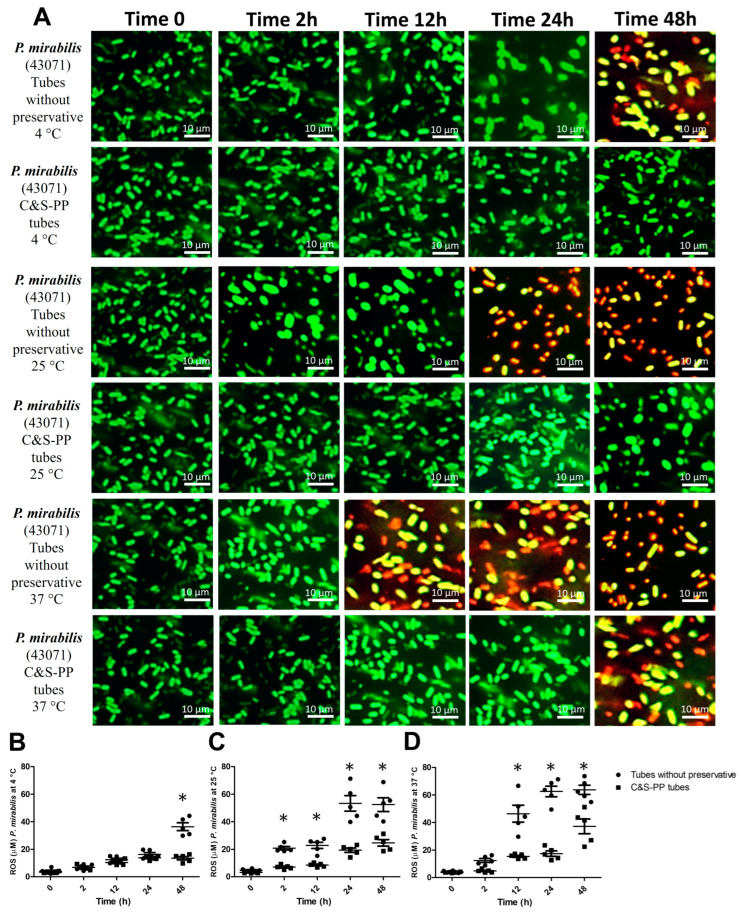
**ROS levels and VBNC *P. mirabilis* at storage times and temperatures with and without preservatives.** (**A**) *P. mirabilis* viable but non-culturable (VBNC); (**B**) *P. mirabilis* ROS level at 4 °C; (**C**) *P. mirabilis* ROS level at 25 °C; (**D**) *P. mirabilis* ROS level at 37 °C. The results shown are the average of five different experiments ± SEM. (*) A two-way ANOVA followed by Bonferroni tests indicates a significant difference regarding the control time, zero minutes (*p* ≤ 0.05). **ROS**, Reactive Oxygen Species.

In *P. mirabilis* at 4 °C, the variable time accounted for 62.4% (F = 86.5) and preservatives for 8.8% (F = 44.9) of the total ROS concentration variance. The ROS concentration differed only at 48 h (103%), and VBNC and dead bacteria were observed (Figure 2A,B) only in the tubes without preservatives. At 25 °C, the preservative affected 25.5% (F = 133.2) of the variance and time incubation, 55.6% (F = 60.5). The ROS concentration increased in tubes without preservatives by 194% (2 h), 167% (12 h), 176% (24 h), and 108% (48 h) (Figure 2C). However, VBNC and dead bacteria were observed at 24 and 48 h in the tubes without preservatives (Figure 2A). Finally, in *P. mirabilis* at 37 °C, preservatives accounted for 22.4% (F = 173.8) and time incubation, 58.4% (F = 74.9) of total ROS variance. The ROS concentration was augmented significantly at 12 (200%), 24 (258%), and 48 h (71%) in tubes without preservatives, and at the same time, VBNC and dead bacteria appeared, while these were observed at 48 h in the C&S-PP tubes (Figure 2A,D).

**Figure 3:**
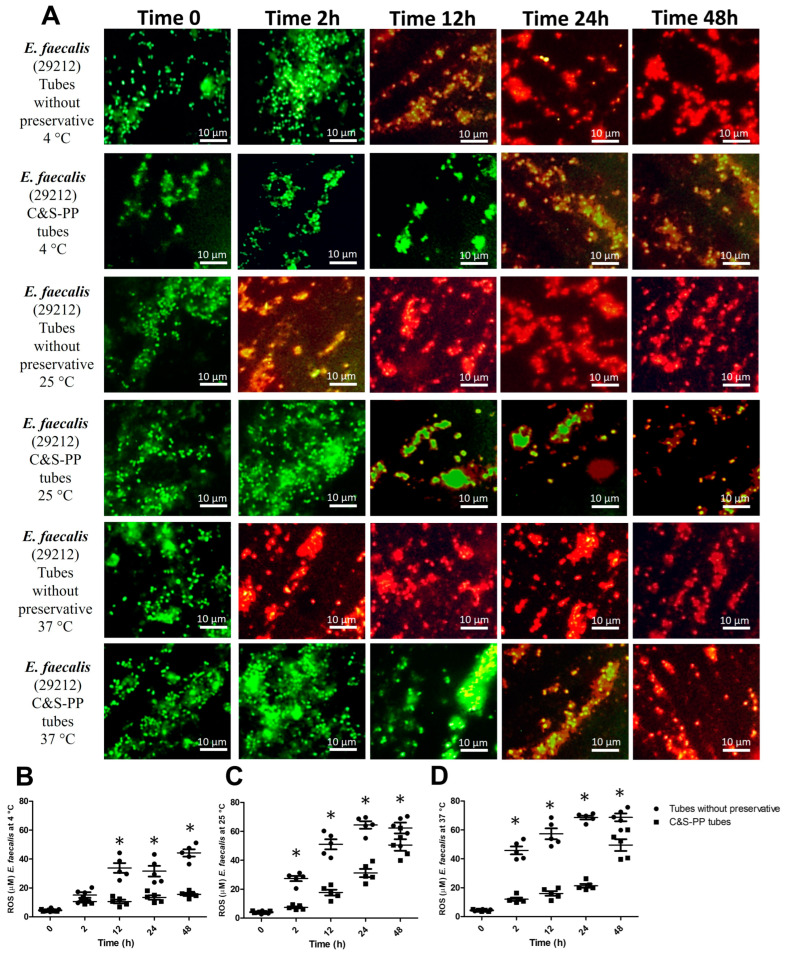
ROS levels and VBNC *E. faecalis* at different storage times and temperatures with and without preservatives. (**A**) *E. faecalis* viable but non-culturable (VBNC); (**B**) *E. faecalis* ROS level at 4 °C; (**C**) *E. faecalis* ROS level at 25 °C; (**D**) *E. faecalis* ROS level at 37 °C. The results shown are the average of five different experiments ± SEM. (*) A two-way ANOVA followed by Bonferroni tests indicates a significant difference regarding the control time, zero minutes (*p* ≤ 0.05). ROS, Reactive Oxygen Species.

For the total ROS concentration variance of *E. faecalis* at 4 °C, the preservative accounted for 18% (F = 318) and the time incubation for 69.3% (F = 126.5). The ROS concentration increased in tubes without preservatives by 218% (12 h), 134% (24 h), and 184% (48 h) (Figure 3B). The VBNC state was observed in tubes without preservatives at 12 h, and in the C&S-PP tubes at 24 and 48 h, while dead bacteria appeared in tubes without preservatives at 24 and 48 h (Figure 3A). The total ROS variance for *E. faecalis* at 25 °C was 32.2% (F = 232.5, preservatives) and 53% (F = 187.2, time incubation). The ROS concentration was significantly high in tubes without preservatives from 2 h until the experiment’s end (Figure 3C). At 25 °C, the VBNC state was observed at 2 h in tubes without preservatives, and from 12 h in the C&S-PP tubes, while a significant number of dead bacteria appeared from 12 h in tubes without preservatives and at 48 h in the C&S-PP tubes (Figure 3A). Finally, the analysis for *E. faecalis* at 37 °C showed that total ROS variance was influenced by the preservatives by 31.3% (F = 48.2), and by time incubation by 43.2% (F = 87.4). Meanwhile, the ROS concentration increased drastically from 2 h in tubes without preservatives, and even in the C&S-PP tubes at 48 h, in this incubation condition (Figure 3D). Therefore, a considerable number of dead bacteria were observed in tubes without preservatives while ROS was augmenting. Likewise, the VBNC state was recognized at 24 h and dead bacteria at 48 h incubation in the C&S-PP tubes (Figure 3A).

**Figure 4:**
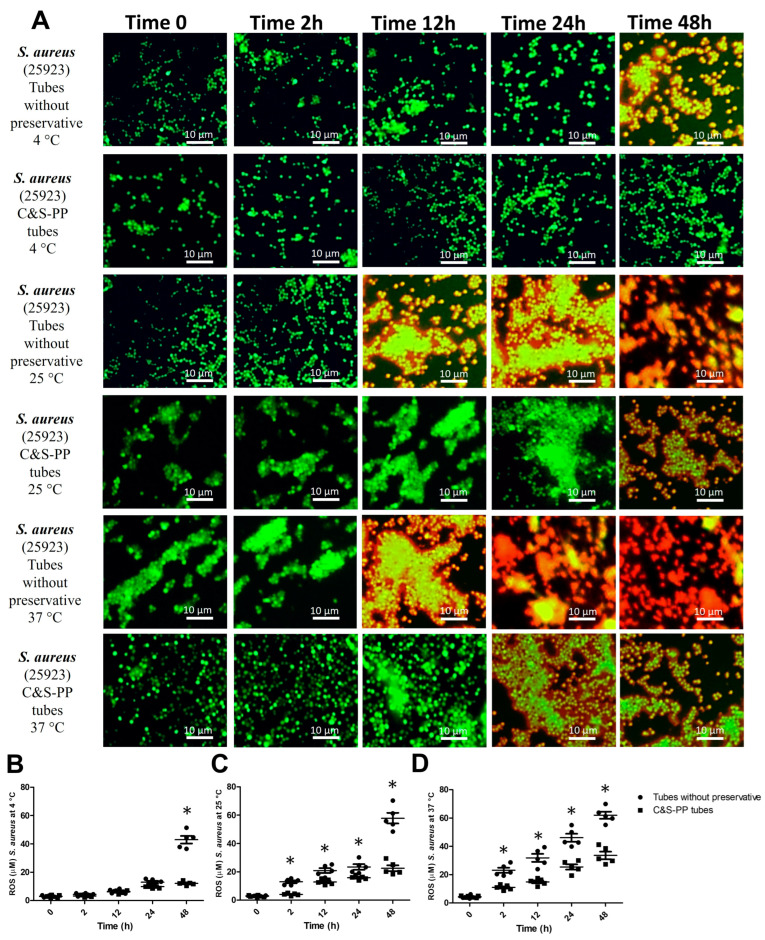
ROS levels and VBNC *S. aureus* at different storage times and temperatures with and without preservatives. (**A**) *S. aureus* viable but non-culturable (VBNC); (**B**) *S. aureus* ROS level at 4 °C; (**C**) *S. aureus* ROS level at 25 °C; (**D**) *S. aureus* ROS level at 37 °C. The results shown are the average of five different experiments ± SEM. (*) A two-way ANOVA followed by Bonferroni tests indicates a significant difference regarding the control time, zero minutes (*p* ≤ 0.05). ROS, Reactive Oxygen Species.

Regarding preservatives, the total ROS concentration variance for *S. aureus* at 4 °C was 9.4% (F = 291), while the time incubation accounted for 61.1% (F = 174.5). The ROS concentration significatively increased at 48 h by 254% (Figure 4B), at the same time that the VBNC state was observed in tubes without preservatives (Figure 4A). At 25 °C, the total ROS variance for *S. aureus* was 14.6% (F = 84.6, preservatives) and 66.2% (F = 175.5, time incubation). At 25 °C, the ROS concentration increased significantly in tubes without preservatives from 2 h by 154%, 63%, 48%, and 157% (Figure 4C). A significative quantity of VBNC bacteria was observed at 12 and 24 h, and dead bacteria at 48 h, in tubes without preservatives, while a low number of VBNC bacteria were observed in the C&S-PP tubes at 48 h (Figure 4A). The analysis for *S. aureus* at 37 °C showed that total ROS variance was influenced by the preservatives by 18.8% (F = 217) and time incubation by 69.6% (F = 143). The ROS concentration increased from 2 h by 112%, 123%, 83%, and 87% in tubes without preservatives (Figure 4D). The VBNC state was observed at 12 h, and bacteria death augmented at 24 and 48 h in tubes without preservatives. Likewise, the VBNC state was increased at 24 and 48 h in the C&S-PP tubes (Figure 4A).

The ROS concentration could positively or negatively affect bacterial growth. Therefore, we performed and analyzed bacteria cultures from each time and temperature. For *E. coli*, it was observed that the number of departure bacteria increased at two hours and 4 °C without preservatives by 14.3%. Meanwhile, in this condition but at 48 h, the number of CFU/mL diminished by 17.6%. The use of preservatives accounted for 1.84% (F = 2.12) and time stored for 49.7% (F = 19.6) of the growth total variance. At 25 °C, *E. coli* showed the same growth behavior, where the preservatives used were 0.22% (F = 0.43) and time stored for 48.4% (F = 18.5) of the growth total variance. At 37 °C, the CFU of *E. coli* from tubes without preservatives increased significantly by 19% (2 h), 24.7% (12 h), 65% (24 h), and 89.7% (48 h). In this condition, the preservatives accounted for 44.4% (F = 281.9) and time stored for 23.1% (F = 69.6) of the growth total variance.

*P. mirabilis* at 4 °C had increased CFU by 29.5% (2 h) and 37.3% (12 h) while diminished by 26.3% (48 h) from tubes without preservatives. Preservatives accounted for 2.3% (F = 4.04) and time stored for 45.4% (F = 49.4) of the growth total variance. At 25 °C, *P. mirabilis* increased CFU by 18.8% (2 h) and 14.3% (12 h), while diminished by 15.5% (24 h) and 21.6% (48 h), from tubes without preservatives. Preservatives accounted for 0.45% (F = 0.42) and time stored for 37.3% (F = 20.2) of the growth total variance. At 37 °C, the CFU of *P. mirabilis* from tubes without preservatives increased significantly by 30.3% (2 h) and diminished by 18.9% (12 h), 24.5% (24 h), and 25.8% (48 h). In this condition, the preservatives accounted for 6.04% (F = 24.4) and the time stored for 37.9% (F = 33.6) of the growth total variance.

Multiple regression analysis for Gram-negative bacteria showed that *E. coli* growth has a high multiple correlation coefficient. However, the determination coefficient was low for strains without preservatives, except those incubated at 37 °C. The critical F value in all cases was minor compared to the calculated F value; thus, the relation between variables was significant. Although the ROS *p*-values were not significant for *E. coli* growth, the time incubation *p*-values were significant at 37 °C. Meanwhile, the analysis for *P. mirabilis* growth showed a minor multiple correlation coefficient, particularly in the C&S-PP tubes at 37 °C. The determination coefficient was very low in all cases. However, the critical F value was below the calculated F value, except for the C&S-PP tubes at 37 °C. The time and ROS *p*-values were not significant for *P. mirabilis* growth (Table 1).

*E. faecalis* at 4 °C had CFU increased by 12.8% (2 h) and diminished by 13.7% (12 h), 24.1% (24 h), and 41.4% (48 h) from tubes without preservatives. Preservatives accounted for 9.8% (F = 22.7) and time stored for 57.7% (F = 58.5) of the growth total variance. At 25 °C, *E. faecalis* CFU significatively diminished by 18.7% (2 h), 23.2% (12 h), 39.4% (24 h), and 43.9% (48 h) from tubes without preservatives. Even in the C&S-PP tubes at 48 h, there was a low bacteria count of 18%. Preservatives accounted for 31.1% (F = 204) and time stored for 49% (F = 52.2) of the growth total variance. At 37 °C, the CFU of *E. faecalis* observed the same growth behavior with and without preservatives. In this condition, the preservatives accounted for 36.2% (F = 98.3) and the time stored for 41% (F = 41.5) of the growth total variance.

*S. aureus* at 4 °C had CFU increased by 7.2% (12 h) and diminished by 12.5% (24 h) and 36.7% (48 h) from tubes without preservatives. Preservatives accounted for 11% (F = 28.2) and time stored for 5.3% (F = 2.5) of the growth total variance. At 25 °C, *S. aureus* CFU increased by 11.3% (2 h) and significatively diminished by 25.2% (12 h), 44% (24 h), and 44.2% (48 h) from tubes without preservatives. In the C&S-PP tubes, at 24 h, the bacteria count was 18% higher. Preservatives accounted for 23% (F = 208.6), and time stored for 42.1% (F = 66.6) of the growth total variance. At 37 °C, the CFU of *S. aureus* diminished by 21.2% (2 h), 44.3% (12 h), 38.3% (24 h), and 47.6% (48 h) in the tubes without preservatives. Meanwhile, in the C&S-PP tubes, bacteria count increased by 13% (2 h) and 22% (12 h), but it was 18% lower at 48 h. In this condition, the preservatives accounted for 41.2% (F = 499.3) and the time stored for 39.5% (F = 62.8) of the growth total variance.

The multiple regression analysis for Gram-positive bacteria showed that *E. faecalis* growth has a high multiple correlation and determination coefficient. The critical F value in all cases was minor compared to the calculated F value. The ROS *p*-value was significant for *E. faecalis* growth at 25 and 37 °C without preservatives. Meanwhile, the time *p*-value was only significant at 25 °C without preservatives. The analysis for *S. aureus* growth also showed an excellent multiple correlation and determination coefficient, except in the C&S-PP tubes at 25 and 37 °C. The critical F value in all cases was also minor regarding the calculated F value. However, the time and ROS *p*-values were not significant for *S. aureus* growth (Table 2).

**Figure 5:**
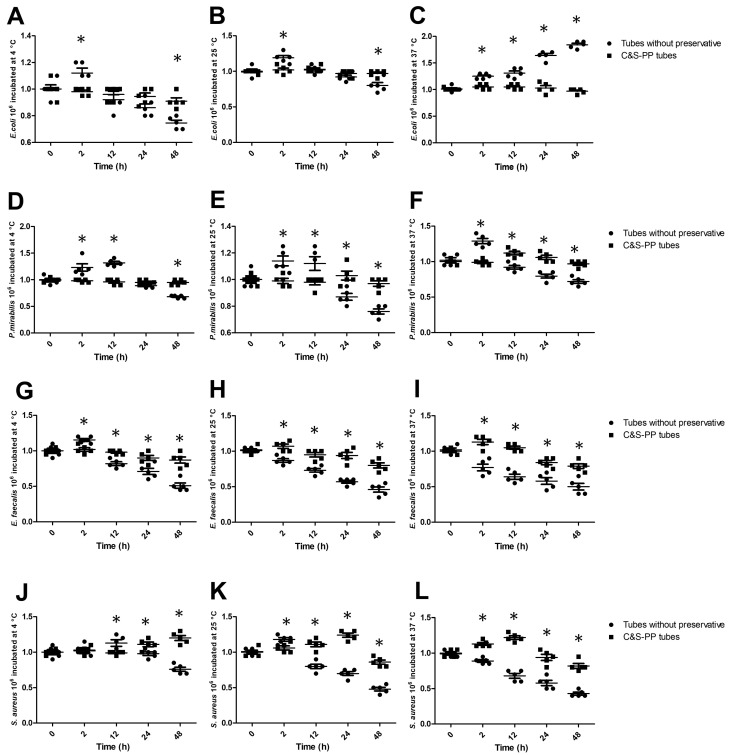
Growth of ATTC strains at different storage times and temperatures with and without preservatives. (**A**–**C**) *E. coli* growth from 4 to 37 °C; (**D**–**F**) *P. mirabilis* growth from 4 to 37 °C; (**G**–**I**) *E. faecalis* growth from 4 to 37 °C; (**J**–**L**) *S. aureus* growth from 4 to 37 °C. The results shown are the average of five different experiments ± SEM. (*) A two-way ANOVA followed by Bonferroni tests indicates a significant difference regarding the control time, zero minutes (*p* ≤ 0.05).

Finally, we corroborated the bacterial growth after storing samples at different temperatures and times. The bacterial counting was performed as colony-forming units per milliliter (CFU/mL), starting from an innocuous 1.0 × 10^6^ cells/mL on each plate. Gram-negative bacteria (*E. coli* and *P. mirabilis*) in C&S-PP tubes at different storage times and temperatures maintained a homogeneous count close to 1.0 × 10^6^ CFU/mL. Meanwhile, *E. coli* in tubes without preservatives increased the CFU/mL number at 2 h and 4 and 25 °C (12% and 19%), but a low count was observed at 48 h at both storage temperatures (25% and 20%), while at 37 °C, the *E. coli* CFU/mL increased progressively and significantly by 24% (2 h), 30% (12 h), 62% (24 h), and 82% (48 h) (Figure 5A–C). *P. mirabilis* stored in tubes without preservatives increased the CFU/mL count at 2 h and at temperatures of 4, 25, and 37 °C (24%, 14%, and 26%), and at 4 h and 4 and 25 °C by 33% and 12%, but a significantly low CFU/mL count at 4 °C and 48 h (31%), at 25 °C and 24 and 48 h (13% and 24%), and at 25 °C from 12 h, by 10%, 22%, and 29% (Figure 5D,E).

On the other hand, storage time and temperature affected the CFU/mL count of Gram-positive bacteria cultures (*E. faecalis* and *S. aureus*) from tubes with and without preservatives. *E. faecalis* stored without preservatives increased the CFU/mL number at 2 h and 4 °C (15%), but a low count was observed from 12 h (18%, 29%, and 49%), while the bacteria stored in C&S-PP tubes at 4 °C had diminished CFU/mL counts by 10% and 13% at 24 and 48 h. At 25 and 37 °C without preservatives, *E. faecalis* presented a low CFU/mL count of 15% and 25% (2 h), 28% and 37% (12 h), 44% and 43% (24 h), and 55% and 51% (48 h). Meanwhile, with preservatives at 25 and 37 °C, *E. faecalis* diminished the CFU/mL count at 24 h by 10% and 16%, and at 48 h by 21% for both temperatures (Figure 5G–I). *S. aureus* stored without preservatives at 4 °C increased the CFU/mL by 13% at 12 h but diminished its count at 48 h by 24%; however, storage in C&S-PP at the same temperature augmented the CFU/mL by 11% and 20% at 24 and 48 h, respectively. The CFU of *S. aureus* stored without preservatives at 25 °C increased at 2 h (17%) and then diminished by 21% (12 h), 31% (24 h), and 52% (48 h). However, at the same temperature but with preservatives, the CFU number augmented at 12 (11%) and 24 h (24%), diminishing at 48 h by 14%. Without preservatives at 37 °C, the CFU number diminished from 2 h by 10%, 31%, 41%, and 57%; however, in C&S-PP tubes, the CFU count increased by 13% and 22% at 2 and 12 h, and diminished by 18% at 48 h (Figure 5J–L). These results confirm and strengthen the predictive analysis in Table 1 and Table 2.

## 4. Discussion

For many years, pre-analytical urinalysis processes have focused on improving technology for quality assurance and reducing diagnostic errors. Technologies for urine preservation are better and, each time, most frequently used to transport the sample to the laboratory or process centers [18,19]. They are mainly used to maintain the number of bacteria and their viability for cultures, optimizing results and treatments. BD Vacutainer™ Urine Culture & Sensitivity Preservative PLUS Plastic Tubes are an option to ensure the pre-analytical state of microbiology cultures; however, little information exists in the literature about how this technology can prevent ROS and the development of the VBNC state regarding the time and temperature of sample storage. In this work, we studied these parameters regarding the growth–die-off dynamic for four common Gram-negative and -positive uropathogenic strains.

In bacteria and other organisms, ROS can be either generated intracellularly due to aerobic metabolism or exogenously from the outside environment because of exposure to oxidative agents [9]. Therefore, bacteria frequently live with ROS in their environment, typically in the form of H_2_O_2_. However, ROS accumulation above the physiological threshold (oxidative stress) has lethal consequences, eliciting cell death [20]. However, the accumulation of ROS itself is bacteriostatic rather than bactericidal [21]. It has been reported for Gram-positive and -negative strains but not specifically for uropathogenic bacteria [22]. Lethal ROS action is derived from a metabolic rate increase and accumulation, which overwhelms the repair of primary damage [20]. Although ROS have a short half-life owing to their high reactivity, they would function as an effector because of their high reactivity and serve as a stress marker [23]. Our results showed a strong interaction of ROS generation dependent on time storage or incubation rather than temperature. This included *E. coli* from 12 h at any temperature without preservatives, although at 37 °C, ROS levels were below that than observed in the other storage temperatures (Figure 1B–D). *P. mirabilis* showed a similar behavior; however, storage temperature plays a critical role regarding storage time, since at 4 °C, ROS increased until 48 h, at 25 °C from 2 h, and at 37 °C increased from 12 h. Interestingly, C&S-PP tubes avoid the ROS increase in Gram-negative bacteria, independent of temperature or storage time (Figure 2B–D).

The C&S-PP tubes prevent metabolic changes in urine analytes and the overgrowth of bacteria by adding stabilizers, such as boric acid, sodium borate, and formate. In our experiment, ATCC strains were established in Culti-Loops Aerobic Set Rehydration Fluid, thereby exempting urine interferences. The results suggest that the preservative combination in C&S-PP tubes reduces the Gram-negative metabolic rate, because the level ROS did not increase despite being stored for a long time at different temperatures, which affects the result with a higher percentage of the total ROS variance. Güneş explains that combining boric acid and sodium borate diminishes the microorganisms’ chemical oxygen demand, reducing their activity [24]. Likewise, a recent study has shown that combining boric acid, sodium borate, and formate can reduce ROS levels in *Pseudomonas aeruginosa* and *E. coli*. The study suggests that combining boric acid, sodium borate, and formate prevents the ROS increase [25].

In the same way, we also analyzed Gram-positive bacteria. *E. faecalis* increased the ROS concentration at 25 °C from 12 h, while at 25 and 37 °C, from 2 h. The statistical analysis showed that this bacterium has a higher dependence on time of storage than temperature and that the preservatives of C&S-PP tubes help to maintain a lower ROS concentration than in bacteria without preservatives, although time of storage and temperature negatively affect it. Meanwhile, *S. aureus* increased ROS concentration until 48 h at 4° C, while at 25 and 37 °C, ROS increased from 2 h, observing a dependence similar to *E. faecalis* regarding time and storage temperature. The C&S-PP tubes do not provide the best preservation conditions for Gram-positive strains, at least for strains analyzed in this work. Therefore, it is possible that Aerobic Set Rehydration Fluid does not provide all the nutrients to Gram-positive bacteria, thereby increasing electron flux through the respiratory chain, triggering ROS production [26]. Particularly, *S. aureus* electron transference from NADH or NADPH to oxygen to produce superoxide generates oxidative stress similar to that encountered during an oxidative burst in phagocytes [27]. A low temperature and preservative use could diminish metabolic requirements for Gram-positive bacteria, and then ROS production decreases regarding strains exposed to environmental or higher temperatures for a long time. However, more research is needed to understand this preservative combination’s exact mechanism of action and potential applications for ROS prevention. Additionally, several studies have shown that bacteria tolerance to adverse environments is associated with reduced respiration, ATP levels, or metabolic activity [26,27,28,29,30]. Studies also showed that starvation increases the number of persister bacteria, which recover from stress [7,28,30]. Therefore, it is possible to induce a VBNC state.

The VBNC state is a survival strategy induced by bacteria in response to adverse conditions; it was previously referred to as a dormancy state. However, differences exist between both, mainly in the VBNC state where the metabolic activity is measurable, but is not in the dormant state. Thus, many medically important bacteria that develop the VBNC state remain in long-term unfavorable and deteriorated conditions, conserving specific features of viable cells but losing culturability with available techniques [14]. This state can be considered an inactive form of life waiting for revival under suitable conditions. Our result showed the presence of VBNC bacteria mainly in tubes without preservatives (yellow and orange bacteria fluorescence), which suggests that ROS and storage time are the inducers of the VBNC state at this stage. This was observed in all analyzed strains (Figure 1 and Figure 4A). In the VBNC stage, bacteria undergo cellular modifications such as energy depletion, altered expression of genes, and DNA replication, which are induced by low nutrient content or oxygen availability and impairment of a particular metabolic pathway [31]. Some *E. coli* strains transit from the culturable to the VBNC state and vice versa due to some organic molecules in the medium, which prolong their survivance and viability [32,33]. Additionally, these VBNC *E. coli* strains resist oxidative stress [34]. It has been informed that *P. mirabilis* in the VBNC state retains its virulence, even in extreme osmotic pressure, starvation, low temperature, and low pH, by stress response genes [35]. The VBNC *E. faecalis* modify their cell wall chemical composition, increasing the number of peptidoglycan oligomers, enzymes involved in their assembly, and autolytic enzymes, making them susceptible to cell death [36,37]. Meanwhile, it has been described that VBNC *S. aureus* displayed mutational inactivation of catalase and superoxide dismutase, rendering the bacterial cells highly sensitive to stress and temperature changes that lead to cell death [38,39,40].

Stress response genes finely regulate the VBNC stage and bacterial death; bacteria with the best and most robust defense mechanisms tolerate a certain amount of non-harmful ROS, resisting fatal events [20,41]. We observed bacterial death (red fluorescence) in *E. coli* at 4 and 25 °C after 12 h storage in tubes without preservatives (Figure 1A), but the count was statistically low only at 48 h, suggesting that the survival mechanisms maintained a culturable bacterial number between 2 and 24 h despite the observed dead bacteria. An ROS increase is associated with a “switch on” of the survival mechanisms. Notably, a slight increase in ROS between 2 and 24 h was observed, which could have induced a major number of CFU before the VBNC stage (Figure 5A). This hypothesis is strengthened because at 37 °C, *E. coli* had a slightly augmented ROS level but the CFU count was higher than the initial innocuous count (Figure 1A,D and Figure 5C). Although this hypothesis must be confirmed by specific experimentation, it has been reported that *E. coli* and other Gram-negative bacteria, such as *P. mirabilis*, possess SOS genes in response to DNA damage that helps them to survive. The SOS constitutive response is crucial for the signal recognition particle pathway that repairs DNA damage and allows cell division as a stressor response [23]. This could also explain the behavior observed in *P. mirabilis*, which presented with bacteria death mainly in tubes without preservatives at long storage times (Figure 2A). Likewise, *P. mirabilis* increased the CFU number at short storage times, but significantly decreased it at long storage times (Figure 5). The multiple regression analysis suggests that ROS and storage time influenced bacteria growth. However, C&S-PP tubes substantially improved this parameter (Table 1).

The specific and adaptive responses are crucial for bacteria survival in adverse conditions. Stress response pathways, such as the heat shock and SOS response pathways, as well as the response to oxidative stress, are widely conserved and exhibit regulatory connections. It has been informed that *E. faecalis* in stress situations expresses the heat shock gene *groES*, *sodA* coding for the oxidative stress-induced superoxide dismutase, and genes of replication, recombination, and repair, coding for DNA polymerase IV. This enzyme is part of the late cellular SOS response induced by DNA damage [42,43]. In the case of *S. aureus*, sixteen genes have been identified under the control of *LexA* and *recA*, both master regulators of the SOS pathway. These genes also play a critical role in several processes related to *S. aureus* pathogenesis, such as antibiotic resistance, dissemination of virulence factors, and diversity in the size and features of colonies. However, both *E. faecalis* and *S. aureus* survivals are strongly influenced by the level and duration of stressors, being more susceptible than Gram-negative bacteria [44,45]. Our results confirmed these findings, since more Gram-positive bacteria cell death was observed in tubes without preservatives than in C&S-PP tubes in short-time storage (Figure 3 and Figure 4A). Additionally, this was confirmed in the bacterial counts (Figure 5). Nevertheless, at 2 h of incubation, the temperature and environmental conditions could activate SOS genes because we observed an increase in the CFU number, which decreased in long-time incubation independent of the temperature, but apparently depends on ROS levels. The multiple regression analysis confirmed that both stressor dependences led to cell death in tubes without preservatives, but ROS concentration presented the best correlation (Table 2).

In summary, our results provide interesting evidence about the effect of temperature and time of storage on ATCC Gram-negative and -positive strains without preservatives and BD Vacutainer™ Urine Culture & Sensitivity Preservative PLUS Plastic Tubes. Gram-negative and -positive strains augmented the ROS concentration time-dependently and were influenced by the storage temperature. Additionally, ROS levels were associated with an increase in the CFU number at short storage times and induction of the VBNC stage at long storage times, alongside cell death and low CFU counts. Gram-positive strains were more affected than Gram-negative bacteria. However, storage in C&S-PP tubes reduces the adverse effects of temperature changes and prolonged storage time. However, this must be corroborated in urine samples under daily work conditions. In conclusion, the C&S-PP tubes minimize the harmful impact of stressors not routinely analyzed on uropathogenic bacteria, optimizing pre-analytical conditions according to the clinical and laboratory standards guidelines.

## Figures and Tables

**Table 1:** Linear regression analysis for Gram-negative strains.

	R	R^2^	F Calculated	Critical Value of F	Time (*p*-Value)	ROS (*p*-Value)
*E. coli* at 4 °C without preservative	0.933	0.741	6.71	0.130	0.966	0.556
*E. coli* at 4 °C C&S-PP	0.997	0.988	169.5	0.006	0.503	0.058
*E. coli* at 25 °C without preservative	0.867	0.502	3.02	0.249	0.411	0.803
*E. coli* at 25 °C C&S-PP	0.786	0.236	1.62	0.382	0.258	0.411
*E. coli* at 37 °C without preservative	0.979	0.917	23.0	0.042	0.043	0.249
*E. coli* at 37 °C C&S-PP	0.945	0.787	8.41	0.106	0.050	0.119
*P. mirabilis* at 4 °C without preservative	0.798	0.273	1.75	0.363	0.514	0.659
*P. mirabilis* at 4 °C C&S-PP	0.852	0.453	2.66	0.273	0.187	0.421
*P. mirabilis* at 25 °C without preservative	0.860	0.481	2.85	0.260	0.368	0.983
*P. mirabilis* at 25 °C C&S-PP	0.836	0.396	2.31	0.302	0.165	0.177
*P. mirabilis* at 37 °C without preservative	0.864	0.494	2.95	0.253	0.650	0.520
*P. mirabilis* at 37 °C C&S-PP	0.418	-0.651	0.21	0.825	0.603	0.628

Multiple regression analysis to assess the relationships between preservatives, temperature, time, and ROS on bacteria growth, with a significance level of *p* ≤ 0.05.

**Table 2:** Linear regression analysis for Gram-positive strains.

	R	R^2^	F Calculated	Critical Value of F	Time (*p*-Value)	ROS (*p*-Value)
*E. faecalis* at 4 °C without preservative	0.943	0.778	7.99	0.111	0.262	0.724
*E. faecalis* at 4 °C C&S-PP	0.949	0.803	9.13	0.099	0.131	0.870
*E. faecalis* at 25 °C without preservative	0.998	0.993	298.5	0.003	0.019	0.013
*E. faecalis* at 25 °C C&S-PP	0.958	0.837	11.3	0.081	0.456	0.646
*E. faecalis* at 37 °C without preservative	0.997	0.989	177.4	0.006	0.084	0.009
*E. faecalis* at 37 °C C&S-PP	0.966	0.866	13.98	0.067	0.082	0.148
*S. aureus* at 4 °C without preservative	0.931	0.735	6.55	0.132	0.490	0.216
*S. aureus* at 4 °C C&S-PP	0.944	0.782	8.17	0.109	0.394	0.940
*S. aureus* at 25 °C without preservative	0.952	0.814	9.75	0.093	0.195	0.459
*S. aureus* at 25 °C C&S-PP	0.762	0.162	1.39	0.419	0.247	0.285
*S. aureus* at 37 °C without preservative	0.982	0.930	27.5	0.035	0.687	0.177
*S. aureus* at 37 °C C&S-PP	0.760	0.156	1.37	0.422	0.500	0.704

Multiple regression analysis to assess the relationships between preservatives, temperature, time, and ROS on bacteria growth, with a significance level of *p* ≤ 0.05.

## Data Availability

Data is contained within the article.
